# Philosophy without natural kinds: a reply to Reydon & Ereshefsky

**DOI:** 10.1007/s13194-024-00594-5

**Published:** 2024-07-27

**Authors:** David Ludwig

**Affiliations:** Wageningen University, Hollandseweg 1, 6706 KN Wageningen, Netherlands

**Keywords:** Natural kinds, Material kinds, Materiality, Grounded functionality, Scientific classification, Ontology

## Abstract

The tradition of natural kinds has shaped philosophical debates about scientific classification but has come under growing criticism. Responding to this criticism, Reydon and Ereshefsky present their grounded functionality account as a strategy for updating and defending the tradition of natural kinds. This article argues that grounded functionality does indeed provide a fruitful philosophical approach to scientific classification but does not convince as a general theory of natural kinds. Instead, the strengths and limitations of Reydon and Ereshefsky’s account illustrate why it is time to move beyond general definitions of “natural kind” and experiment with new philosophical frameworks.

Categories, classifications, concepts, nomenclatures, ontologies, and taxonomies are of foundational importance for scientific practice. In philosophy of science, the “tradition of natural kinds” (Hacking, 1991) has dominated many debates about conceptual structures of science. Epistemological concerns about the explanatory fruitfulness of scientific concepts, methodological debates about classificatory and taxonomic practices, and metaphysical controversies about nature of scientific ontologies all have relied heavily on the assumption that empirical inquiry leads to the discovery of natural kinds that in turn shape conceptual structures of science.

While the tradition of natural kinds is very much alive (Kendig, 2016; Khalidi, 2023) and continues to inspire excellent research, it has also become the target of growing criticism. The complaints are numerous: The tradition of natural kinds is historically steeped in essentialist assumptions that are incompatible with the conceptual complexity of science (Dupré, 2002). It has been accused of misleading through the assumption of one general demarcation criterion between “the natural” and “the non-natural” (Ludwig, 2018a). It has been criticized as sidelining normative and political concerns about scientific concepts (Brigandt, 2022; Ludwig, 2023). The appeal to “naturalness” has also been challenged as expandable for philosophical analysis (Hacking, 2007; Papale & Montminy, 2023) and as relying on an unconvincing metaphysics of kind realism (Chakravartty, 2023).

While each of these complaints would deserve a separate discussion, the growing criticism reflects that the future of natural kinds has become very much contested. I’ve added to some of the complaints above but my discontent with tradition of natural kinds is also motivated by the wider sense that it has limited philosophical debates about conceptual structures of science through an excessively narrow focus. As paradigmatically expressed in Lewis’ (1999, 3) appeal to an “elite minority”, the tradition of natural kinds has often been grounded in a conceptual elitism that divides the space of human concepts into a scientific elite minority that “carves nature at its joints” and an ordinary peasant majority that lacks naturalness. This elitism has not only led to the quick dismissal of “folk concepts” as metaphysically flawed but also to the neglect of vast swaths of fruitful scientific concepts that do not meet narrow criteria of naturalness.

While philosophers of science have aimed to moderate this elitism by paying increased attention to “gray zones” between the natural and the non-natural, the tradition of natural kinds continues to be a major source of parochial attitudes that have largely divorced philosophy of science from interdisciplinary research on classificatory practices in fields such as anthropology, communication sciences, education, history, journalism, law, science studies, sociology, or policy studies. This disconnect is most clearly on display in debates about contested scientific categories such as “anthropocene”, “biodiversity”, “disability”, “gender”, “indigeneity”, “innovation”, “intelligence”, “mental disorder”, “neurodiversity”, “pandemic”, “poverty”, “obesity”, “race”, or “sustainability”. While philosophers have increasingly turned their attention to such concepts, the tradition of natural kinds often constraints interdisciplinary engagement. Reminiscent of 20th-century divisions between a philosophically relevant “context of justification” and a merely sociological “context of discovery” (Schickore & Steinle, 2006), an isolated focus on narrow criteria for naturalness pushes much of the social complexity of classificatory practices to the periphery. The result is that the tradition of natural kinds often drives constrained analyses of such practices while much of the corresponding empirical research does not even take note of philosophical demarcation disputes about the definition of natural kinds.

While it's easy to complain about philosophical traditions and their historical baggage, it is much more difficult to propose feasible alternatives. My article “From Naturalness to Materiality: Reimagining Philosophy of Scientific Classification” (Ludwig, 2023) aimed to move beyond critique along three dimensions: First, challenging the exclusionary legacy of Lewis’ “elite minority” through an inclusive focus on materiality. Traditional cases of natural kinds such as chemical elements or biological species are material but so are household items or social institutions, allowing for a much broader discussion about the many ways in which concepts are shaped by material realities. Second, easing metaphysical anxieties about the abandonment of the tradition of natural kinds. The alternative to the tradition of natural kinds is not an unrestricted constructivism or relativism but creates space for novel debates about the entanglement of conceptual and material structures. Third, alternative coalition building as “materiality” and “materialism” have become widely theorized across social sciences and humanities. While the tradition of natural kinds has separated philosophy of science from these expansive debates about materiality (e.g. Barad, 2007, Tuin & Dolphijn, 2013), a turn towards material kinds would allow for a more inclusive debate that brings diverse intellectual traditions together.

## Capturing grounded functionality

In their commentary “Ethnobiological Kinds and Material Grounding: Comments on Ludwig”, Reydon and Ereshefsky (2024) develop a thoughtful defense of the tradition of natural kinds and particularly of their own proposal of operationalizing naturalness in terms of “grounded functionality”. While being sympathetic to many complaints about the tradition of natural kinds, Reydon and Ereshefsky argue that “natural kind” remains an important notion for understanding classificatory practices and propose an account that can be specified in terms of two core requirements: First, natural kinds need to be grounded in the sense that they “should depend on an aspect of the world rather than merely on human interests” (2024, 4). Grounding distinguishes natural kinds from purely conventional kinds or kinds that are falsely assumed to exist without referring to anything in reality. However, not all grounded kinds should qualify as natural kinds. For example, some categories may be grounded in artificial ways (think of Goodman’s (1955) grue and bleen) or simply lack fruitfulness for scientific inquiry. The second functionality condition therefore requires that a “classification should be judged by how well it functions in achieving the aims it is posited for” (2024, 4).

Reydon and Ereshefsky provide careful responses to many complaints that have been leveraged against the tradition of natural kinds. For example, their framing avoids essentialist assumptions by acknowledging the many ways in which grounding and functionality can be achieved. By exploring this diversity, Reydon and Ereshefsky are also not restricted to Lewis’ “elite minority” but their account allows for explorations across a wide range of scientific programs in both natural and social sciences. Acknowledging the roles of non-epistemic values in determining functionality, their account also accounts for the importance of normative and political concerns in classificatory practices rather than sidelining them as irrelevant for questions of naturalness. The upshot of Reydon and Ereshefsky’s discussion is therefore that iconoclastic critiques of the tradition of natural kinds are based on a greatly exaggerated problem diagnosis (see also Conix & Chi, 2021). Instead of abandoning the notion of natural kinds and proposing a substitute such as material kinds, we really just need a level-headed reform that relieves the tradition of natural kinds from some of its historical baggage.

My goal here is not to criticize grounded functionality but rather to capture it for the purposes of my campaign against the tradition of natural kinds. Reydon and Ereshefsky indeed develop nuanced and thoughtful insights, showing how grounding and functionality operate across a wide range of scientific disciplines and convey important aspects about fruitful classificatory practices. While their account illustrates that a lot of valuable work continues to be produced within the tradition of natural kinds, its main limitation derives from being framed within this tradition. Although grounded functionality has a lot of potential for nuanced analysis, its framing as a general account of natural kinds leads back to scholastic demarcation disputes that limit its usefulness. Rather than rejecting the grounded functionality account, my goal here is therefore to capture it from the tradition of natural kinds and to argue that it is much more convincing when standing on its own.

As I have argued elsewhere (Ludwig, 2018a), the tradition of natural kinds tends to create scholastic demarcation disputes by trying to draw one general line between the “natural” and the “non-natural” despite mounting evidence of a plurality of relevant factors for fruitful classificatory practices. Recent definitions of natural kinds have appealed, for example, to homeostatic property clustering (Wilson et al., 2007), stable property clustering (Slater, 2015), nodes in causal networks (Khalidi, 2018), pure projectability (Valero, 2023), conceptual stability across epistemic actors (Franklin-Hall, 2015), and grounded functionality (Ereshefsky & Reydon, 2023). Each of these accounts conveys relevant insights about classificatory practices but each of them becomes inadequate when claiming priority over the others as the definite and general demarcation criterion for what should count as “natural”. It is simply not credible that one of these dimensions is preferable across all classificatory practices and across all scientific disciplines. Instead of leading to meaningful unification, general definitions of “natural kind” are therefore always vulnerable to counter examples that expose the definition as too broad or too narrow for some purposes.

In the case of grounded functionality, I tried to illustrate this problem with case studies from ethnobiology (Ludwig, 2023). Ethnobiology provides a rich entry point for thinking about the diversity of classificatory practices around the world. It also provides a rich entry point for exploring the diversity of academic concerns about classification: Some ethnobiological studies have very restricted concerns about understanding cross-cultural convergences of classifications, employing a narrow understanding of natural kinds for which grounded functionality turns out to be too broad. Other ethnobiological studies aim to understand cross-cultural diversity of biological classifications beyond science, employing a broad understanding of natural kinds for which even grounded functionality turns out to be too narrow.

The main goal of Reydon and Ereshefsky’s (2024) commentary is to show that these objections are misguided and that their account is able to navigate this diversity of ethnobiological concerns about classificatory practices. While this debate concerns specific questions about the merits of grounded functionality, our disagreement also conveys broader lessons for debates about the future of natural kinds. I wholeheartedly agree that grounded functionality can provide a valuable lens for engaging with diversity of classificatory concerns in ethnobiology. I wholeheartedly disagree that grounded functionality provides a unified account of natural kinds. I therefore aim to embrace grounded functionality while capturing it for my own purposes: thinking about classificatory practices beyond the failed project of one general definition of “natural kind”.

## Narrow naturalness

Reydon and Ereshefsky aim for an inclusive account of natural kinds that captures the many different ways in which grounding and functionality can be achieved. Such an inclusive definition is indeed helpful for philosophers of science who aim to understand diverse classificatory practices across heterogeneous scientific disciplines. However, this does not mean that grounded functionality is suitable as a general definition of “natural kind”. While their inclusive account is useful for understanding diverse classificatory practices in science, it is too broad for many other projects that rely on the notion of natural kinds.

In the case of ethnobiology, I illustrated this limitation with a research tradition that appeals to natural kinds to explain cross-cultural convergence of biological classifications (see also Ludwig, 2018b for a review of this tradition). In this research tradition, natural kinds are understood as objective “discontinuities in nature” (Hunn, 1977) that reflect “the structure of nature itself” (Berlin et al., 1966, 275), are recognized by vastly different cultures, and therefore produce classificatory universals around the world. In contrast with this restrictive use of “natural kind,” grounded functionality includes many local classifications that are unique to specific cultures but still satisfy grounding and functionality relative to the purposes of a particular community. The upshot is that grounded functionality (just as all other competitor definitions of “natural kind”) works well for some purposes (e.g. explaining diversity of classificatory practices in science) while being inadequate for other purposes (e.g. explaining cross-cultural convergence of classificatory practices).

Reydon and Ereshefsky respond to this objection by distinguishing between three levels of analysis. First, local communities classify the biological world. Second, ethnobiologists identify some of these (e.g. only cross-culturally stable) classifications as “natural kinds”. Third, philosophers analyze concepts of both local communities and ethnobiologists. Reydon and Ereshefsky insist that their account is located on the third level and should not be expected to match all the uses of “natural kinds” on the second level. Instead, grounded functionality is supposed to allow for an analysis of concepts on both the first and second level. According Reydon and Ereshefsky, my ethnobiological example does therefore not provide a counterexample but can actually be analyzed through the grounded functionality account by telling “us – in this case philosophers studying how ethnobiology works – why the distinction between natural kinds and other kinds in this particular context of scientific practice is successful” (2024, 5).

I appreciate this response as it indeed nicely shows that grounded functionality provides a productive lens for analyzing diverse concepts of both local communities and scientists. However, I never meant to challenge this productivity but rather the suitability of grounded functionality as a general definition of “natural kind”. And when it comes to the latter issue, the authors simply presuppose what is contested when writing “the task of a philosophical account of natural kinds is to help philosophers make sense of classificatory practices” (2024, 3).

The point of my counterexample was to show that this is only one of many tasks that is perfectly legitimate but cannot claim priority over other tasks such as making sense of cross-cultural stability of classifications. Distinguishing between different levels does not solve this problem as diversity of uses of “natural kind” on the second level of empirical research is intertwined with diversity of uses at the third level of philosophical analysis. For example, ethnobiological research on cross-cultural convergence was itself deeply influenced by philosophical debates about species realism and metaphysical claims about objective “discontinuities in nature” that transcend cultural relativity of classificatory practices (see Ludwig, 2018b).

While Reydon and Ereshefsky suggest that there is one core task of philosophical accounts of natural kinds, my example was meant to show that there are different tasks that also motivate different philosophical accounts. For example, Franklin-Hall (2015) proposes an account of natural kinds “categorical bottlenecks” that are recognized by diverse epistemic actors. Her account does not only match ethnobiological concerns with cross-cultural convergence (Ludwig, 2016, 2017) but also reflects long-standing philosophical debates about “joints in nature” that limit cross-cultural relativity and instead make natural kinds “to some degree line up with one another” (Franklin-Hall, 2015, 932) across cultures and epistemic practices. Philosophers who approach debates about natural kinds with epistemic concerns about cross-cultural convergence or metaphysical questions about “joints in nature” will not be satisfied by grounded functionality because they are looking for something more restricted such as Franklin-Hall “categorical bottlenecks”. In contrast, philosophers who approach debates about natural kinds with concerns about the diversity of fruitful classificatory practices in science are very well served by grounded functionality and will find “categorical bottlenecks” too restrictive.

Grounded functionality provides a very useful lens for engaging with diverse classificatory practices but Reydon and Ereshefsky set their account up for failure by suggesting that it fulfills the main or singular “task of a philosophical account of natural kinds” (2024, 3). No matter whether we look at the second level of empirical sciences or the third level of philosophy, the problem was from the very beginning that “natural kind” is mobilized for a lot of different tasks that respond to equally different concerns. Some of them are nicely captured by grounded functionality, others are not. Rather than rejecting the grounded functionality account, I therefore want to suggest liberating it from the failed program of formulating one general definition of “natural kind”. The result would not be a failure of Reydon and Ereshefsky’s proposal but rather allow it to shine in the context of its own goals: explaining the diversity of fruitful classificatory practices in science.

## Broad naturalness

While ethnobiological debates about cross-cultural convergence show that grounded functionality is sometimes too broad, other ethnobiological contexts suggest that the account can also be too restrictive for engaging the diversity of local classifications of the biological world. In a previous publication, for example, Reydon and Ereshefsky (2022) discussed whether local classifications of whales as fish would qualify as natural kinds. Such classifications appear grounded in the sense of depending “on an aspect of the world rather than merely on human interests” (2024, 4). Whales and fish share a wide range of phenotypic and behavioral features that are the product of co-adaptation to aquatic life. Putting them together also seems functional for many local communities that are not interested in phylogenetic relations but may recognize relevant behavioral, ecological, or morphological similarities among polyphyletic groups in the context of daily livelihood practices such as fishing.

Still, Reydon and Ereshefsky (2022) argued that such classifications do not identify natural kinds as their account “asks for well-supported assumptions, and for assumptions that can be tested and confirmed in the first place. Here, the onus is on relevant areas of science to explicate what aspects of the world a classification connects to, to explain how these aspects of the world enable the classification to achieve the epistemic and non-epistemic aims that are in focus, and to do so in a way that is testable.” According to the authors, local classifications of whales as fish do not count as natural kinds “as long as the relevant community does not provide a well-confirmed account that connects these aims to aspects of nature” (2022, 3).

While such additional requirements of well-confirmed and testable accounts may work in analyzing classificatory practices within science, I’ve argued (Ludwig, 2023) that it limits the usefulness of the account for ethnotaxonomic studies. Ethnotaxonomists aim to understand the structure of local classifications, the grounding of such classifications in community knowledge about the biological world, and their functionality for local practices such as environmental management or farming (Ludwig, 2016). Suggesting that such classifications only identify natural kinds once communities theorize their classifications through well-confirmed and testable accounts akin to scientific research seems artificial and limits the usefulness of their account for engaging classificatory practices outside of academia. It’s not like communities are not able to produce justificatory accounts but they are not always the focus and making them a necessary condition exports criteria from scientific research to other domains that are more adequately discussed on their own.

In their response, Reydon and Ereshefsky suggest that it is sufficient that “*someone* – a local community of users *or* the relevant academic community studying that local community – provides such an account” (2024, 3). This appears to constitute a small modification of their proposal that leads to a more charitable interpretation of their previous example of “folk classification of cetaceans as fish” (2022). Even if a community does not provide a well-confirmed and testable account of this classification it may still satisfy Reydon and Ereshefsky’s criteria as long as someone else has developed such an account. And indeed, ethnobiologists (Souza & Begossi, 2007) and even philosophers (Dupré, 1999) have done precisely that by exploring the grounding and functionality of classifications of whales as fish.

While this liberalization addresses some of my previously raised concerns, it still remains awkward to apply Reydon and Ereshefsky’s account to ethnobiological studies of local classifications. It seems to suggest that local classificatory practices often fail to identify natural kinds until some academic researcher enters the community to confirm and theorize this classification. It seems rather artificial to think of local kinds *becoming* natural kinds only once they have been theorized by some external researcher. It also seems to build an unnecessary hierarchy into the notion of “natural kind” by withholding that label from communities who often do not formulate what professional scientists would recognize as a “well-supported account”. To put it in polemic terms: It is both artificial and paternalistic to claim that some community failed to identify natural kinds until Dupré (1999) came along and provided a theoretical justification of the inclusion of whales in the category of fish.

Again, the problem is not the grounded functionality account but rather its framing as a general account of natural kinds. Just as the account was not designed to address metaphysical worries about cross-cultural convergence, it was also not designed to analyze the classificatory practices of local communities. Instead, it was designed to analyze classificatory practices across diverse scientific disciplines. It does this job exceptionally well. Problems only appear once the account is positioned as a general theory of natural kinds that is claimed to be applicable across all contexts. It is only through this overgeneralization that Reydon and Ereshefsky’s requirement of well-confirmed and testable accounts becomes awkward because it is clearly formulated to capture scientific practices and there is no need to impose it on local and everyday classificatory practices. Again, it seems to me that grounded functionality would be nothing but liberated by seeking distance from demarcation disputes around one authoritative definition of “natural kind” with the inevitable sequence of counterexamples, redefinitions, more counterexamples, and more redefinitions. Grounded functionality can very well stand on its own.

## Moving on without natural kinds

While the preceding discussion has addressed some rather specific concerns about grounded functionality, they are very much illustrative of the wider state of natural kind debates. Even if the tradition of natural kinds carries historical baggage, a lot of valuable work continues to be produced within this tradition (see also Khalidi, 2023 for a helpful review). The work of Reydon and Ereshefsky is exemplary of this as it provides a very valuable lens for analyzing classifications in scientific practice. The same applies to philosophical discussions about issues such as property clustering, causal unification, or projectability that all provide valuable and largely complementary insights about the structures of classificatory practices but are usually pitched against each other in an unproductive competition for the formulation of one general demarcation criterion.

The value of grounded functionality would not be diminished but actually much clearer if Reydon and Ereshefsky would develop their account independently from such demarcation debates about the true mark of naturalness. I’d very much welcome grounded functionality as an ally in the project of reimagining philosophy of classification beyond the tradition of natural kinds. A more productive debate beyond natural kinds would embrace the plurality of valuable accounts of thinking about fruitful classifications in (and beyond) science. Grounded functionality can provide valuable insights just as research on property clustering, causal unification, projectability, or materiality.

It is in this sense that I would also like to position grounded functionality as complementary rather than competing with my own proposal of a theory of material kinds (Ludwig, 2023). While there is some overlap between our proposals, they also serve different functions. First, Reydon and Ereshefsky develop an insightful account of classificatory practices in science while my goal was broader to also understand the materiality of everyday kinds that may lack functionality in science. Second, my account of material kinds was formulated with the metaphysical aim of easing anxieties about abandoning the tradition of natural kinds. Rather than collapsing into some untenable constructivism or relativism, theorizing materiality provides a nuanced entry point for understanding dynamics between conceptual and material structures. Even if these metaphysical concerns are not at the center of Reydon and Ereshefsky’s account, I’d assume that they are very much complementary to their explorations of grounded functionality.

Finally, the notion of material kinds also aims to build new coalitions with the expansive debates about materiality outside of analytic philosophy and is therefore also part of my response to the authors’ worries that the “terms ‘natural’ and ‘natural kind’ are [too] deeply entrenched in both philosophy and science” (2024, 12) for abandonment. My suspicion is that if we look at “philosophy and science” without the selection bias of analytic philosophy of science, we’d find talk about materiality to be at least as deeply entrenched as talk about naturalness and therefore our best bet for building more diverse intellectual coalitions. Abandoning the tradition of natural kinds also opens new intellectual horizons that connect to a broader range of research projects beyond the often excessively narrow focus of philosophers on criteria for naturalness. While the debate about one general definition of “natural kind” has become largely divorced from interdisciplinary inquiries about both epistemological and social complexity of classificatory practices in science, philosophers could contribute valuable tools to such debates. I would greatly appreciate the grounded functionality account as an ally in exploring new avenues beyond the limitations of the tradition of natural kinds.
